# Dexamethasone potentiates chimeric antigen receptor T cell persistence and function by enhancing IL-7Rα expression

**DOI:** 10.1016/j.ymthe.2023.12.017

**Published:** 2023-12-22

**Authors:** Ashlie M. Munoz, Ryan Urak, Ellie Taus, Hui-Ju Hsieh, Dennis Awuah, Vibhuti Vyas, Laura Lim, Katherine Jin, Shu-Hong Lin, Saul J. Priceman, Mary C. Clark, Lior Goldberg, Stephen J. Forman, Xiuli Wang

**Affiliations:** 1 Department of Hematology and Hematopoietic Cell Transplantation, City of Hope, Duarte, CA 91010, USA; 2 Division of Cancer Epidemiology and Genetics, National Cancer Institute, Bethesda, MD, 20892, USA; 3 Department of Clinical Translational Project Development, City of Hope, Duarte, CA 91010, USA

**Keywords:** dexamethasone, chimeric antigen receptor, CAR, glucocorticoid, CAR T cells, IL-7Rα

## Abstract

Dexamethasone (dex) is a glucocorticoid that is a mainstay for the treatment of inflammatory pathologies, including immunotherapy-associated toxicities, yet the specific impact of dex on the activity of CAR T cells is not fully understood. We assessed whether dex treatment given *ex vivo* or as an adjuvant *in vivo* with CAR T cells impacted the phenotype or function of CAR T cells. We demonstrated that CAR T cell expansion and function were not inhibited by dex. We confirmed this observation using multiple CAR constructs and tumor models, suggesting that this is a general phenomenon. Moreover, we determined that dex upregulated interleukin-7 receptor α on CAR T cells and increased the expression of genes involved in activation, migration, and persistence when supplemented *ex vivo*. Direct delivery of dex and IL-7 into tumor-bearing mice resulted in increased persistence of adoptively transferred CAR T cells and complete tumor regression. Overall, our studies provide insight into the use of dex to enhance CAR T cell therapy and represent potential novel strategies for augmenting CAR T cell function during production as well as following infusion into patients.

## Graphical abstract

**Figure:**
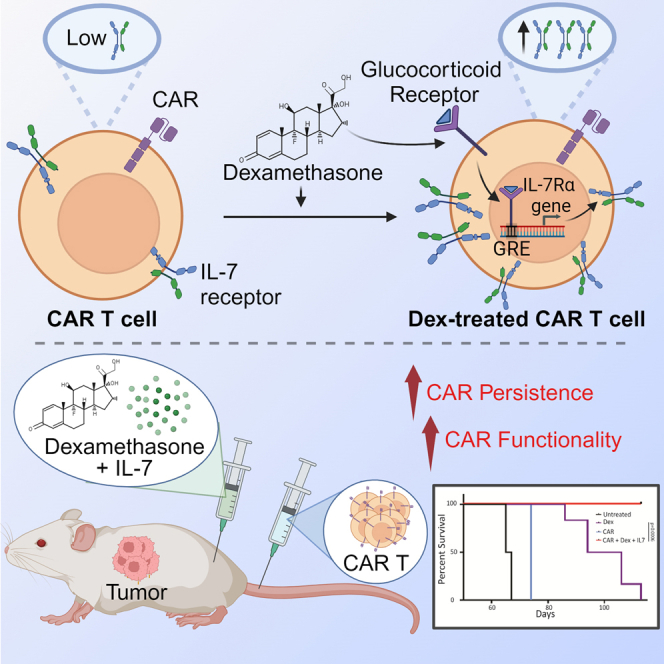

## Teaser

Wang and colleagues unveiled the debated role of dex in CAR T cell therapy. Contrary to concerns, the study demonstrated no adverse effects on CAR T cell expansion or function. Furthermore, dex elevated IL-7Rα expression, enhancing responsiveness to IL-7 and consequently improved CAR T cells' anti-tumor activity.

## Introduction

Glucocorticoids (GCs) are a class of steroids that are routinely used to treat hyper-inflammatory conditions, including the toxicities related to chimeric antigen receptor (CAR) T-cell therapy. The binding of GCs to a GC receptor (GR) causes GR dimerization and translocation to the nucleus, where GRs modulate the expression of genes that contain a GC response element.^1^ In general, GCs are not routinely given to patients receiving CAR T cells, except to treat severe therapy-related toxicity (i.e., cytokine release syndrome [CRS] and/or immune effector cell-associated neurotoxicity syndrome) because GCs could potentially dampen CAR T cell activity and persistence.^2^^,^^3^ Recent clinical data suggests that GCs, including the synthetic GC dexamethasone (dex), do not affect proliferation or persistence of infused CAR T cells,^4^^,^^5^ suggesting that the effects of GCs on CAR T cells are complex and not fully characterized.

GCs affect the expression of genes related to T cell function and survival,^6^^,^^7^ including the upregulation of the cytokine receptor interleukin-7 receptor α (IL-7Rα or CD127) in both murine and human T cells.^1^^,^^6^ Signaling through IL-7Rα activates the JAK-STAT pathway, which plays a critical role in T cell homeostatic proliferation and memory formation.^8^ IL-7Rα is highly expressed on naive and central memory T cells but found in low levels on effector memory T cells.^8^^,^^9^^,^^10^^,^^11^ Clinically, CAR T cell products with memory-like features, including high levels of IL-7Rα, coupled with high serum IL-7 following lymphodepletion prior to CAR T cell infusion, positively influence patient outcomes, including progression-free survival and overall survival,^12^^,^^13^ suggesting that IL-7/IL-7Rα signaling may enhance the potency of CAR T cell therapy. However, CAR T cells downregulate IL-7Rα upon activation and *ex vivo* expansion during the manufacturing process,^11^^,^^14^^,^^15^ which may impair *in vivo* persistence. We hypothesized that GCs such as dex could be leveraged to increase the level of IL-7Rα on CAR T cells if given strategically during the manufacturing process, which may overcome the challenge of insufficient CAR T cell persistence.

Here, we pre-clinically evaluated the use of dex during CAR T cell manufacturing and as an adjuvant during CAR T cell therapy. We determined the optimal dose schedule of dex during manufacturing that upregulated IL-7Rα on CAR T cells, improving their responsiveness to IL-7, but did not impact CAR T cell expansion, phenotype, or function *in vitro*, or anti-tumor activity in multiple *in vivo* tumor models of both hematologic and solid malignancies. In the adjuvant setting, tumor-bearing mice treated with dex and IL-7 post-CAR T cell infusion had superior survival outcomes compared with mice treated with dex or IL-7 alone after CAR T cell infusion in both dex-sensitive and dex-insensitive tumor models. Overall, this work suggests that the use of GCs during CAR T cell manufacturing and therapy may lead to superior outcomes through the enhancement of IL-7Rα signaling and, therefore, identifies a potential benefit of GCs alongside CAR T cell therapy.

## Results

### *Ex vivo* dex did not affect CAR T cell expansion, phenotype, or function

**Figure 1:**
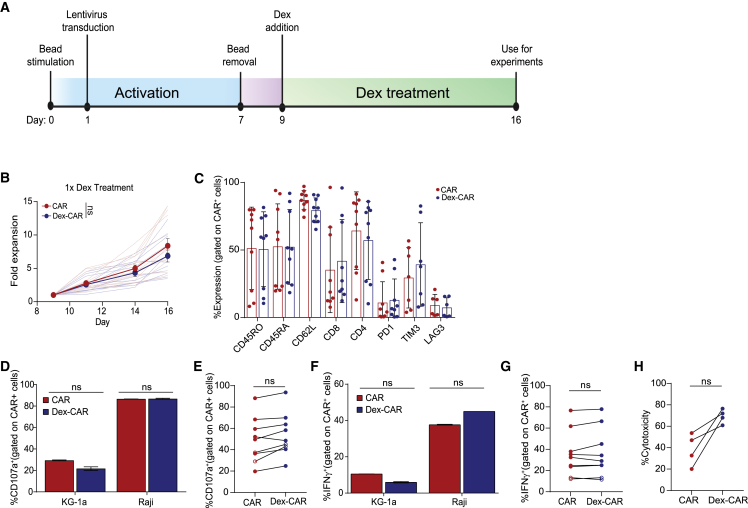
Dex does not affect CAR T cell expansion or effector functions (A) PBMCs were activated with anti-CD3/anti-CD28 beads on day 0, transduced with lentivirus on day 1, followed by bead removal on day 7. A single 1-μM dex dose was added to the culture on day 9. Dex-treated CAR (dex-CAR) and non-dex-treated CAR T cells (CAR) were analyzed on day 16. (B) Expansion of CAR T cells for 14 HDs, grown in the presence or absence of a single dose of 1 μM of dex. Thick lines are averages over all HDs for each condition. Fold changes to pre-treatment are presented. (C) 1 μM dex-CAR T cells or non-dex-treated CAR were stained with indicated immune receptors and analyzed them with a flow cytometer, and the percentages of positive cells are presented. Representative data of CAR T cells from nine different HDs are presented. (D) Representative data of CD107a-positive degranulation 1 μM dex-CD19-CAR T cells after 6 h co-culturing with CD19^+^ target Raji cells, measured via flow cytometry. Representative data of CAR T cells from nine different HDs are presented. Myeloid leukemia KG-1a was used as a negative control. (E) Cumulative degranulation data comparing CAR to dex-CAR from nine different HDs, including both CD19 (closed circles) and CS1 (open circles) CAR T cells are presented. The same donor points are connected. (F) Representative data of intracellular IFN-γ secretion by 1 μM dex-CD19-CAR T cells after overnight stimulation with Raji cells. Representative data of CAR T cells from nine different HDs are presented. (G) Cumulative data from nine different HDs, including both CD19 (closed circles) and CS1 (open circles) CAR T cells are presented. The same donor points are connected. (H) Cytotoxicity, measured with a long-term killing assay after 24 h of co-culture of CAR or dex-CAR with Raji target cells, from 2 different HDs, is presented. Data are shown as mean ± SEM with p values by Mann-Whitney tests. ns, not significant.

We manufactured CAR T cells in the presence of 1 μM dex, a dose selected based on previous literature.^16^ We added dex on day 9 of CAR T cell production, when activation beads were removed to decrease additional manipulation of the CAR T cells during the manufacturing process (Figure 1A). A single dose of 1 μM dex on day 9 did not significantly impact fold expansion compared with untreated CAR T cells, which was consistent across 14 separate healthy donors (HDs) (Figure 1B). Furthermore, a single dose of 1 μM dex did not affect expansion of either CD19-28z CAR or CS1-41bbz CAR T cells (Figure S1A), suggesting that this observation is not CAR-specific and may be generalizable. To determine whether dex induces phenotypic changes on CAR T cells, we evaluated CAR T cells (N = 9 HDs) manufactured with (dex-CAR) or without (CAR) 1 μM dex by flow cytometry. We observed comparable levels of memory markers (CD45RO, CD62L, and memory stem marker CD45RA), ratios of CD4^+^ to CD8^+^ cells, and levels of exhaustion markers (PD1, TIM3, and LAG3) on dex-CAR vs. untreated CAR T cells (Figure 1C), which was consistent regardless of CAR specificity (Figures S1B and S1C).

To determine whether 1 μM dex during manufacturing affects CAR T cell effector function, we evaluated the ability of CD19-specific dex-CAR T cells to degranulate and produce cytokines upon co-culture with CD19^+^ target Raji cells. Both untreated and dex-CAR T cells had similar levels of degranulation and cytokine production (Figures 1D–1G), as determined by CD107a and interferon (IFN)-γ positivity, respectively, which was consistent across 7 HDs (Figures 1E and 1G). This phenomenon was not CAR-specific. The effector functions of both CD19-28z and CS1-41BBz CAR T cells were unaffected by a single dose of 1 μM dex (Figures 1E and 1G). Moreover, untreated CAR and dex-CAR T cells induced similar levels of cytotoxicity when co-cultured with CD19^+^ tumor cells (Figure 1H).

A recent report found that high doses (10 μM) and multiple dosages of dex can affect CAR T cells.^17^ Thus, we tested a range of dex concentrations, including both high (10 μM) and low (0.1 μM) doses, with either a single or multiple dex treatments (Figure S2A). We found that low concentrations of dex (0.1–1 μM) did not impact CAR T cell growth, as expected (Figures S2B–S2D). However, 10 μM dex decreased fold expansion, yet did not decrease CAR T cell degranulation (Figure S2E). We confirmed that our results were not influenced by ethanol (dex solvent), which did not affect CAR T cell fold expansion or phenotype, even when given multiple times during manufacturing (Figures S2F and S2G). Together, these data suggest that the negative influence of dex on expansion at high doses is not coupled to effector function and is not influenced by the ethanol in the dex formulation. Building on these findings, we selected 1 μM dex as the optimal dosage for subsequent experiments.

**Figure 2:**
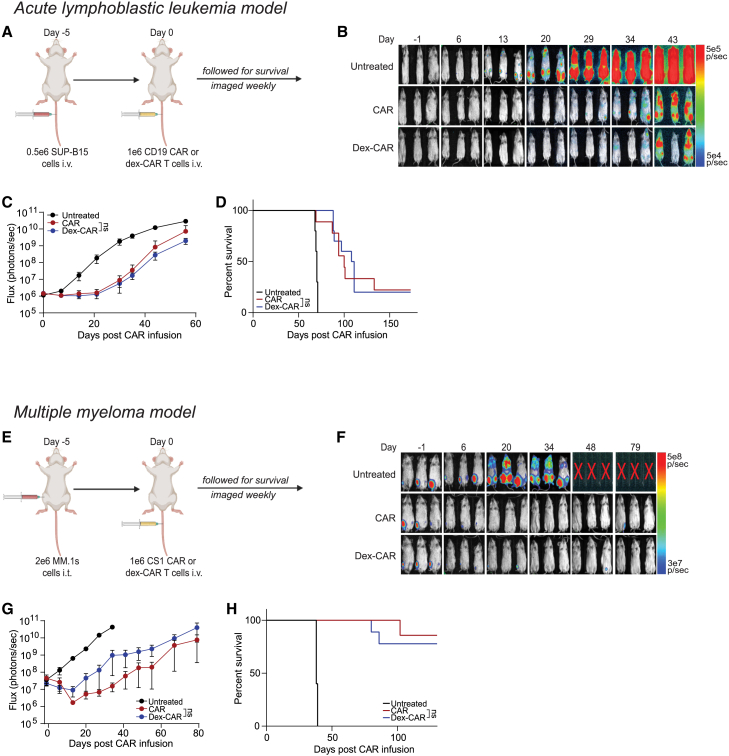
Dex-treated CAR T cells exhibit antitumor activity comparable to untreated CAR T cells (A) 0.5 × 10^6^ ALL (SUP-B15) cells expressing GFP and firefly luciferase (GFPffluc^+^) were inoculated into NSG mice i.v. After confirmation of engraftment, 1 × 10^6^ dex-CAR or non-dex-treated CD19-CAR T cells were adoptively transferred into tumor-bearing mice i.v. (B and C) Biophotonic imaging was used for tumor signal monitoring and tumor burden was measured in flux (photons/sec) by bioluminescent imaging and evaluated weekly. Statistical significance was analyzed on the final day of imaging (day 56). (D) Overall survival was monitored, and Kaplan-Meier curves were generated. (E–H) Experiments were conducted using a MM (MM.1S) model. (E) 2 × 10^6^ MM cells (MM.1S) were injected i,t. into NSG mice on day −5. After confirmation of engraftment, 1 × 10^6^ dex-CAR or non-dex-treated CS1-CAR T cells were adoptively transferred into tumor-bearing mice i.v. (F and G) Biophotonic imaging was used for tumor signal monitoring and tumor burden was measured in flux (photons/s) by bioluminescent imaging and was evaluated weekly. Statistical significance was analyzed on the final day of imaging (day 79). (H) Kaplan-Meier survival curve. For all experiments, N = 10 mice/group. Data are shown as mean ± SEM with p values by Mann-Whitney tests or log-rank (Mantel-Cox) for Kaplan-Meier curves. ns, not significant.

We then tested the antitumor activity of CAR T cells manufactured with 1 μM dex in xenograft mouse models. In our acute lymphoblastic leukemia (ALL) model (Figure 2A), we engrafted 0.5 × 10^6^ SUP-B15 ALL cells intravenously (i.v.) into NSG mice, treated with 1 × 10^6^ CD19-dex-CAR or untreated CD19-CAR T cells, and evaluated tumor growth and mouse survival. CD19-CAR and CD19-dex-CAR T cells were statistically similar in their anti-tumor activity and tumor control, translating to equally extended mouse survival past untreated mice in our ALL model (Figures 2B–2D). This observation was consistent in a second xenograft model of multiple myeloma (MM) using CS1-targeting CAR T cells (Figure 2E). In this model, we injected 2 × 10^6^ MM.1S MM cells intratibially (i.t.) into NSG mice followed by 1 × 10^6^ CS1-CAR T cells i.v., as previously described.^18^ CS1-dex-CAR T cells were comparable with CS1-CAR T cells in delaying tumor growth and extending mouse survival when compared with untreated mice, though insignificant to each other (Figures 2F–2H). Together, these data suggest that dex given during manufacturing does not affect CAR T cell function, either *in vitro* or *in vivo*.

### *In vivo* dex administration did not affect CAR T cell potency

**Figure 3:**
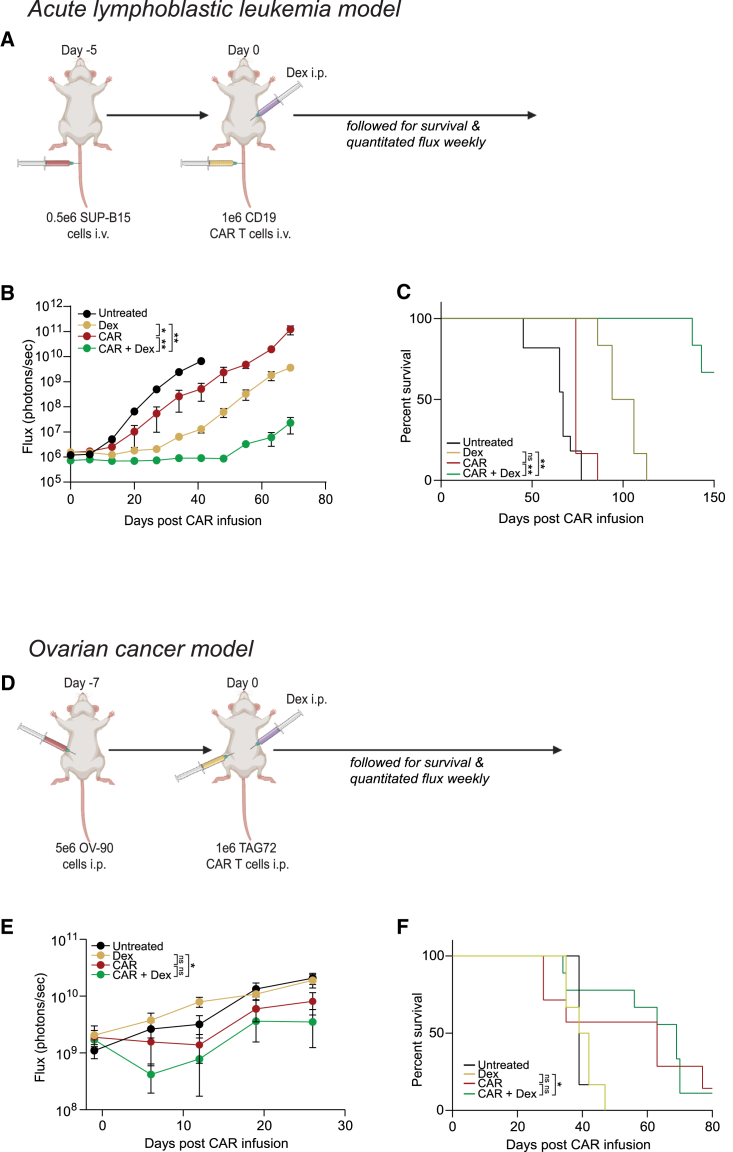
Dex treatment *in vivo* does not affect adoptively transferred CAR T cell potency (A) 0.5 × 10^6^ SUP-B15 tumor cells expressing GFP and firefly luciferase (GFPffluc^+^) were inoculated into NSG mice i.v. After confirmation of engraftment, 1 × 10^6^ CD19-CAR T cells were adoptively transferred into tumor-bearing mice i.v. and dex injections (i.p.) were given. (B) Tumor signal was monitored weekly using biophotonic imaging and tumor burden was measured in flux (photons/sec) by bioluminescent imaging. Statistical significance was analyzed based on the final imaging day of the CAR-only group (day 63). (C) Overall survival was monitored, and Kaplan-Meier survival curves were generated. (D) Experiments were conducted using an ovarian cancer tumor model. 5 × 10^6^ OV-90 ovarian cancer were injected i.p., on day −7. We injected 1 × 10^6^ TAG72-CAR T cells i.p. on day 0, and dex injections were initiated on day 0. (E) Tumor signal was monitored weekly using biophotonic imaging and tumor burden was measured in flux (photons/sec) by bioluminescent imaging. Statistical significance was analyzed on the final day of imaging (day 26). (F) Overall survival was monitored, and Kaplan-Meier survival curves were generated. For all experiments, N = 5–11 mice/group. Data are shown as mean ± SEM with p values by Mann-Whitney tests or log-rank (Mantel-Cox) for Kaplan-Meier curves. ∗p < 0.05; ∗∗p < 0.01; ns, not significant.

Building on our *ex vivo* dex results, we interrogated whether direct injection of dex into mice impacts the efficacy of CAR T cells manufactured without dex. Using our SUP-B15 mouse model (Figure 3A), as previously described,^19^ we treated mice engrafted with SUP-B15 cells with CD19-CAR T cells i.v. and 10 mg/kg dex^16^ intraperitoneally (i.p.). Mice treated with dex alone or CAR T cells alone were used as controls. The combination of CAR T cells and dex was superior in delaying tumor growth and extending mouse survival compared with either CAR T cells or dex alone (Figures 3B and 3C). However, dex alone had some anti-tumor activity, which is likely due to the intrinsic sensitivity of ALL cells to dex.^20^ Because ALL cells are sensitive to dex, we used a dex-insensitive ovarian cancer model. Dex-resistant ovarian tumor model, OV-90, which is an aggressive analog of Skov3, was treated with TAG72-targeting (TAG72)-CAR T cells.^21^^,^^22^ We engrafted mice with 5 × 10^6^ OV-90GFPffluc^+^ cells i.p.,^23^ treated with 1 × 10^6^ TAG72-CAR T cells i.p. with or without dex (Figure 3D). As expected, dex alone had no effect in this tumor model and CAR T cells alone had better tumor control and extended mouse survival compared with untreated mice (Figure 3E). There was equivalent tumor growth and mouse survival in mice treated with CAR T cells alone and in combination with dex (Figures 3E and 3F), suggesting that dex did not impact the *in vivo* activity of CAR T cells. Together, our data demonstrate that dex, when administered during manufacturing or as a combination therapy *in vivo*, does not inhibit CAR T cell activity.

### Dex upregulated IL-7Rα on CAR T cells

**Figure 4:**
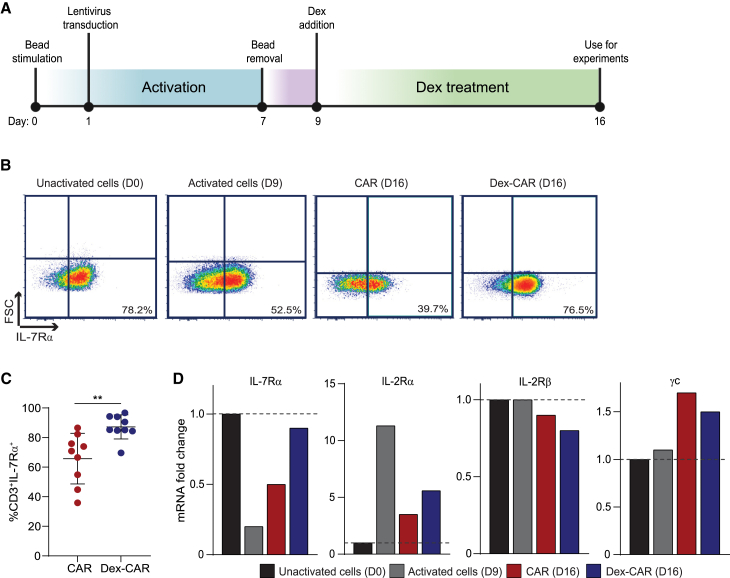
Dex upregulates IL-7Rα on CAR T cells (A) PBMC-derived CD19-dex-treated and non-dex-treated CAR T cells were generated with one treatment of 1 μM dex. (B) IL-7Rα surface expression on PBMC-derived CD19-CAR T cells prior to transduction (unactivated cells; day 0), after transduction (activated cells; day 9), non-dex-treated CAR (CAR; day 16), or dex-treated CAR (dex-CAR; day 16); Representative data of CAR T cells from 14 different HDs are presented. (C) Accumulative data of IL-7Rα^+^CD3^+^ CAR on dex-CAR compared with non-dex-treated CAR T cells (gated CAR T cells) on day 16; representative data of CAR T cells from nine different HDs are presented. (D) Levels of mRNA fold change of IL-7Rα, IL-2Rα, IL-2Rβ, and common gamma chain (γc) on unactivated (day 0), activated (day 9), and CAR or dex-CAR (day 16) PBMC-derived CD19-CAR T cells, treated with and without dex, were determined by NanoString gene analysis, and normalized to un-activated (day 0) cells. N = 2 HD. Dotted lines represent a ratio of 1. Data are shown as mean ± SEM with p values by Mann-Whitney tests. ∗∗p < 0.01.

Dex increases the expression of IL-7Rα on activated T cells.^1^^,^^6^ Considering CAR T cells are activated by CD3/CD28 bead stimulation during manufacturing, we assessed the impact of dex on IL-7Rα expression on CAR T cells. We monitored IL-7Rα expression during CAR manufacturing as follows: before activation (D0), after transduction and bead removal (D9), and 7 days after culture with or without dex (D16) (Figure 4A). Consistent with our previous study,^11^ approximately 80% of non-activated T cells expressed IL-7Rα on day 0, while only approximately 50% of T cells were IL-7Rα positive after activation/transduction on day 9. On day 16, approximately 80% of dex-CAR T cells expressed IL-7Rα, while IL-7Rα expression decreased to approximately 40% in untreated CAR T cells (Figure 4B). This observation of increased IL-7Rα expression on CAR T cells treated with dex was consistent over 9 HDs (Figure 4C) and was independent of either CAR construct (Figs. S1B and S1C) or dex concentration (Figure S3A). We further assessed the ability of dex to increase IL-7Rα on extensively expanded and differentiated, EBV-specific central memory (T_cm_) and effector memory (T_em_) T cells, which are populations with inherent low/no expression of IL-7Rα. We expanded EBV-specific T_cm_ and T_em_ cells *ex vivo* for 3 months by rapid expansion method,^24^ then treated them with or without dex, and analyzed the surface expression of IL-7Rα. Both T_cm_ and T_em_ EBV-specific T cells had increased IL-7Rα expression upon dex treatment (Figure S3B), suggesting that dex can upregulate IL-7Rα expression on activated T cells regardless of differentiation state.

To determine the global impact of dex on gene expression, we analyzed CAR T cells treated with or without dex by NanoString gene analysis. Using a pathway score analysis, we found that dex upregulated expression of genes in pathways related to activation, migration, persistence, and chemokine production relative to untreated CAR T cells (n = 2), which is consistent with an enrichment of memory T cells.^10^ In contrast, pathways related to apoptosis and T cell receptor diversity were downregulated by dex (dashed lines indicate z > +1.96 or z < –1.96) (Figure S4). We identified 33 genes that were differentially regulated by dex, including the upregulation of activation-related genes including IFNGR2, VAV3,^25^ DDIT4,^26^ and IL-31,^27^ migration genes CXCR4^28^ and AREG,^29^ as well as genes that promote memory T cell formation, including IL-7Rα, TNFRSF11A, and ACVR1C.^30^ Dex downregulated effector function-associated genes such as GZMA, tumor necrosis factor, and exhaustion gene programmed cell death ligand 1. To understand whether dex affected other gamma chain receptor genes beside IL-7Rα, we analyzed IL-2 receptor alpha, beta, and shared common gamma chain gene expression after dex treatment and calculated fold change of each gene by normalizing expression levels on days 9 and 16 to that of non-activated cells (D0). We found that IL-7Rα was the only gamma chain cytokine receptor gene upregulated by dex (Figure 4D), suggesting that the effect of dex was specific to IL-7Rα.

### Exogenous IL-7 enhanced *in vivo* efficacy of dex-CAR T cells

**Figure 5:**
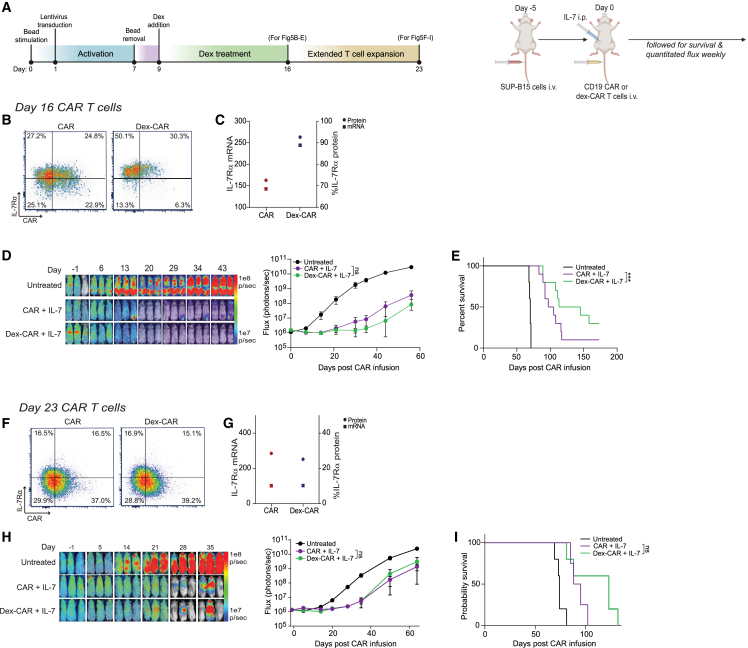
Dex-related effects on CAR T cells are reversible (A) CAR T cells were generated and treated with 1 μM dex once on day 9 (dex-CAR) and removing dex on day 16. The cultures were extended to day 23 and cells were collected for *in vitro* and *in vivo* experiments. 0.5 × 10^6^ SUP-B15 cells expressing GFP and firefly luciferase (GFPffluc+) were inoculated into NSG mice i.v. on day −5 and 1 × 10^6^ CD19-CAR or dex-CAR T cells were given i.v. on day 0. CHO-IL7 cells i.p. injection started on day 0 and then every other day. (B) IL-7Rα expression from dex-CAR or untreated CAR input cells on day 16. (C) Representative data of IL-7Rα mRNA (left axis, square symbols) and protein expression (right axis, circle symbols) in CAR T cells treated with or without 1 μM dex on day 16. mRNA levels were determined with NanoString analysis and protein expression was analyzed by flow cytometry. (D and E) Day 16 dex-CAR and non-dex-treated CD19-CAR T cells were adoptively transferred into tumor-bearing mice i.v., after confirmation of tumor engraftment. Biophotonic imaging was used for tumor signal monitoring and tumor burden was measured in flux (photons/sec) by bioluminescent imaging and evaluated weekly. N = 10 mice/group. Statistical significance was analyzed on the final day of imaging (day 55). (E) Overall survival was monitored, and Kaplan-Meier survival curves were generated. (F) IL-7Rα expression from dex-CAR or untreated CAR on day 23. (G) Representative data of IL-7Rα mRNA (left axis, square symbols) and protein expression (right axis, circle symbols) in CAR T cells treated with or without 1 μM dex on day 23. mRNA levels were determined with NanoString analysis and protein expression was analyzed by flow cytometry. (H) Day 23 dex-CAR and non-dex-treated CD19-CAR T cells were adoptively transferred into tumor-bearing mice i.v., after confirmation of tumor engraftment. Biophotonic imaging was used for tumor signal monitoring and tumor burden was measured in flux (photons/sec) by bioluminescent imaging and evaluated weekly. N = 5 mice/group. Statistical significance was analyzed on the final day of imaging (day 79). (I) Overall survival was monitored, and Kaplan-Meier survival curves were generated. For all experiments, N = 5–10 mice/group. Data are shown as mean ± SEM with p values by Mann-Whitney tests or log rank (Mantel-Cox) for Kaplan-Meier curves. ns, not significant. ∗∗p < 0.01.

IL-7Rα is a key receptor related to CAR T cell persistence, and several strategies have been evaluated to upregulate or overexpress IL-7Rα on CAR T cells.^31^^,^^32^^,^^33^ To determine whether sustained IL-7Rα expression on dex-CAR T cells conferred improved biological activity, we treated ALL-bearing mice with dex-CAR (Figure 5A) in combination with exogenous IL-7 administered through i.p. injections of human (hu)IL-7-secreting CHO cells. As expected, IL-7Rα expression on dex-CAR was higher than that of untreated CAR T cells at both protein and RNA levels (N = 2 HD) (Figures 5B and 5C). Mice received six injections, every 48 h, of 10 × 10^6^ 8,000 rads-irradiated huIL-7-secreting CHO cells, which we confirmed increased serum levels of huIL-7 (Figure S5). Both the CAR and dex-CAR T cell groups had significantly better anti-tumor activity than the untreated group (Figure 5D). Moreover, mice treated with dex-CAR in combination with IL-7 had significantly prolonged survival compared with mice given untreated CAR T cells and IL-7, with 4 of 10 mice versus 1 of 10 mice alive after 180 days, respectively (Figure 5E).

One concern for persistent expression of IL-7Rα on CAR T cells is the potential for uncontrolled persistence and/or tumorigenesis.^34^^,^^35^ Therefore, we tested whether IL-7Rα levels remained high after withdrawal of dex in an extended culture to day 23 (Figure 5A). In contrast with dex-CAR T cells expanded to day 16 from the same donor, there was no difference in IL-7Rα expression on day 23-dex-CAR and untreated CAR T cells at the protein or RNA level (Figures 5F and 5G). In our *in vivo* ALL model, both day 23-CAR groups had better efficacy than untreated mice, but the combination of dex-CAR and IL-7 no longer prolonged survival over untreated CAR T cells and IL-7 (Figures 5H and 5I). Moreover, patterns of gene expression in dex-CAR T cells by NanoString analysis reverted to levels similar to untreated CAR T cells by day 23 when compared with day 16 cells generated from the same donor (Figure S4). Together, these data support the reversibility of dex-mediated changes in gene expression, including upregulation of IL-7Rα.

### *In vivo* combination of dex and IL-7 cytokine enhanced the efficacy of CAR T cells

**Figure 6:**
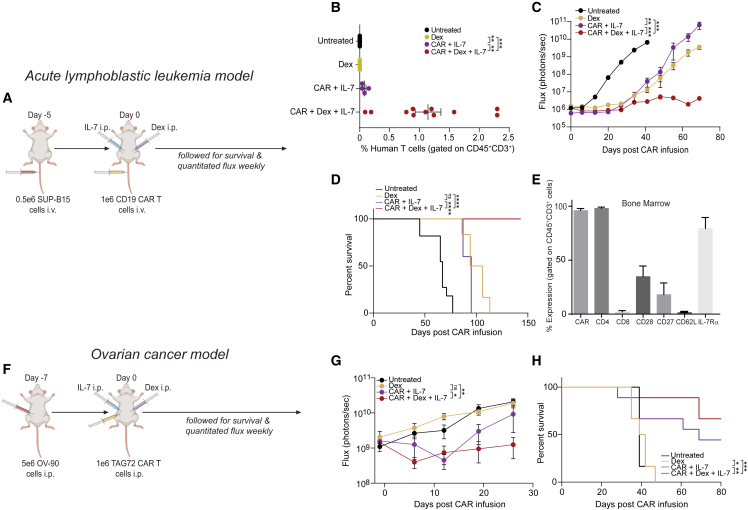
Dex in combination with huIL-7 enables CAR T cells to eliminate tumor *in vivo* (A) T_n/mem_-derived CD19-CAR T cells were activated with anti-CD3/anti-CD28 beads on day 0, transduced with lentivirus on day 1, and beads removed on day 7. CAR T cells were either treated with a single dose of dex on day 9 or three doses on days 9, 11, and 13. Simultaneously, IL-7 was supplemented on days 9, 11, 13. 0.5 × 10^6^ SUP-B15 tumor cells expressing GFP and firefly luciferase (GFPffluc^+^) were inoculated into NSG mice i.v.). After confirmation of engraftment, 1 × 10^6^ CD19-CAR T cells were adoptively transferred into tumor-bearing mice i.v. The mice were injected i.p. with 8,000 rads-irradiated human IL-7-producing CHO cells (5 × 10^6^) and i.p. dex (10 mg/kg), as described. (B) Human T cells (CD45^+^CD3^+^) were collected from the mice by retroorbital bleeding on day 25 following CAR T cell injections and human T cells were quantified via flow cytometer. (C) Tumor signal was monitored weekly using biophotonic imaging and tumor burden was measured in flux (photons/sec) by bioluminescent imaging. Statistical significance was analyzed on the final day of imaging (day 69). (D) Overall survival was monitored, and Kaplan-Meier survival curves were generated. (E) CAR and immune receptors on human T cells in bone marrow collected at euthanasia (day 150) were analyzed with flow cytometry (dex+CAR+IL-7 group). Percentages based on gated CD45^+^CD3^+^ human T cells are presented. (F) Experiments were conducted using an ovarian cancer tumor model. We injected 5 × 10^6^ OV-90 ovarian cancer cells i.p. on day −7. 1 × 10^6^ TAG72-CAR T cells were injected i.p. on day 0, and dex and IL-7 injections were initiated on day 0, as described. (G) Tumor signal was monitored weekly using biophotonic imaging and tumor burden was measured in flux (photons/sec) by bioluminescent imaging. Statistical significance was analyzed on the final day of imaging (day 26). (H) Overall survival was monitored, and Kaplan-Meier survival curves were generated. For all experiments, N = 5–11 mice/group. Data are shown as mean ± SEM with p values by Mann-Whitney tests or log-rank (Mantel-Cox) for Kaplan-Meier curves. ∗p < 0.05; ∗∗p < 0.01; ∗∗∗p < 0.001; ∗∗∗∗p < 0.0001; ns, not significant.

We previously found that dex as an adjuvant did not decrease the *in vivo* function of CAR T cells (Figure 3). Although the combination of CAR+dex initially diminished tumor in our ALL model, this treatment ultimately led to disease rebound (Figure 3B). We, therefore, asked whether the addition of exogenous IL-7 to this combination group could augment CAR T cell function, leading deeper remission. We treated ALL bearing mice with a single infusion of 1 × 10^6^ CD19-CAR T cells, 10 mg/kg dex, and huIL-7-secreting cells i.p. (Figure 6A). To assess dex+IL-7-mediated CAR T cell expansion, we collected blood by retroorbital bleeding on day 25 after CAR T infusion and measured CAR T cell levels by flow cytometry. Mice receiving the combination of CAR T cells+dex+IL-7, but not the CAR+IL-7 group, had circulating human T cells on day 25 (Figure 6B), which corresponded with a significantly lower tumor burden over time (Figure 6C). Moreover, 100% of mice receiving CAR T cells+dex+IL-7 in combination, but not CAR+IL-7 alone, survived to 150 days, when the experiment was terminated without evidence of remaining ALL (Figures 6C and 6D). Human T cells (CD45^+^CD3^+^) in the bone marrow harvested at euthanasia from mice given the combination of CAR+dex+IL-7 were almost entirely CAR positive and retained high expression of IL-7Rα (Figure 6F). Although mice treated with CAR T cells+dex alone had prolonged survival, tumors started to return by day 45 (Figure S6). Moreover, CAR+dex+IL-7-treated mice had superior survival, no tumor relapse, and were also the only treatment group to have CAR T cell presence in retro-orbital blood collection on day 25 (Figure 6B) and in the bone marrow at euthanasia on day 150 (Figure 6E). These CAR T cells extracted at euthanasia also still had sustained high levels of IL-7Rα. All of these results together suggest that the increase in IL-7Rα, induced by dex, allowed for enhanced CAR T cell function and persistence in context of exogenous IL-7.

We repeated these experiments with the dex-resistant ovarian mouse model (OV-90), as described (Figure 6F).^23^ We injected tumor-bearing mice with a single infusion (i.p.) of TAG72-CAR T cells in combination with 10 mg/kg dex and huIL-7-secreting cells i.p. As in our ALL model, the combination of CAR+dex+IL-7 conferred significantly better tumor control than the CAR+IL-7 group (Figure 6G). Although mice in all treatment groups eventually had tumor growth, the combination of CAR+dex+IL-7 led to significantly prolonged survival over either the CAR+IL-7 group or the CAR+dex group (Figure 6H and S6). In contrast with the ALL model, dex alone did not affect survival of mice with OV-90. Because the combination of CAR+dex+IL-7 increased survival over mice given only CAR+IL-7, it is likely that both dex and IL-7 together with CAR T cells contribute to the survival benefit in this model, rather than either dex or IL-7 alone (Figure S6). Due to the specific trafficking of CAR T cells delivered with i.p. injection in this model, we could not detect CAR T cells in the blood or bone marrow upon euthanasia. Collectively, these data confirm that dex-mediated IL-7Rα upregulation on CAR T cells increases their responsiveness to IL-7, which allows for CAR T cells to have improved anti-tumor activity in the presence of IL-7.

## Discussion

Although dex is a well-established immunosuppressive agent, recent clinical observations determined that patients treated with dex post-CAR T cell infusion had comparable numbers of circulating CAR T cells as patients who did not receive dex.^4^^,^^5^ We found that low concentrations (1 μM) of *ex vivo* dex did not impact CAR T cell growth, phenotype, or function, which confirms the findings of Brummer and colleagues,^17^ who reported that *ex vivo* dex only impacts CAR T cells at supraphysiological concentrations (Figure 1 and S2). Furthermore, we found that *ex vivo* or *in vivo* administration of dex did not impact CAR T cell function in multiple cancer models (Figures 1–3 and S2), and that dex may synergize with CAR T cells in dex-susceptible tumors such as ALL (Figure 3 and S6). While these data imply that dex does not inhibit CAR T cells, it also opens novel potential for combination therapies using CAR T cells and dex in dex-susceptible diseases, such as ALL and MM.^36^^,^^37^

Our data highlight the ability of dex to upregulate genes promoting memory formation, activation, and trafficking that may enhance CAR T cell efficacy (Figure S4). Of those genes, we focused on the pro-proliferation and pro-survival gene IL-7Rα, which is upregulated at both the mRNA and protein levels, regardless of CAR design, dex concentration, or T cell differentiation state (Figures 4, 5, S1, S3, and S4). IL-7Rα is a key driver of CAR T cell persistence *in vivo*, however, activation using anti-CD3 during *ex vivo* culture of CAR T cells results in downregulation of IL-7Rα (Figure 4).^38^^,^^39^^,^^40^ To overcome this problem, we and others previously investigated constitutively expressing active IL-7 receptor that confers exogenous, cytokine-independent, cell-intrinsic STAT5 signaling and observed improved adoptive T cell therapies in preclinical models.^31^^,^^32^^,^^33^ However, these methods are not readily translatable to the clinic due to the potential for uncontrolled T cell proliferation and tumorgenicity.^34^^,^^35^ Thus, establishing clinically applicable methods of retaining and/or controlling upregulation of IL-7Rα expression on CAR T cells, such as using dex, are favorable.

We investigated whether *ex vivo* administration of dex as a manufacturing supplement or as an adjuvant through *in vivo* administration in combination with IL-7 may benefit CAR T cell function. In both methods, increased IL-7Rα conferred enhanced responsiveness to exogenous IL-7 and persistence (Figures 5, 6, and S6). We further demonstrated that upregulation of IL-7Rα on CAR T cells by dex is transient and reversible (Figure 5), which may be a safety feature when CAR T cells are given to patients. In addition, we show as an adjuvant, dex in combination with CAR T cells and IL-7 led to long-term tumor control.

The presence of IL-7 in patients has been mathematically modeled and shown to improve CAR T cells, likely due to its role in homeostatic T cell proliferation and survival.^41^^,^^42^ Lymphodepleting chemotherapy can increase serum concentration of IL-7, which is associated with the length of progression-free survival in the context of CAR T cell therapy.^13^ Thus, it is possible that CAR T cells with high IL-7Rα expression following *ex vivo* dex could have superior persistence and efficacy in patients who have high serum IL-7 after lymphodepletion. Other proposed strategies to increase IL-7 concentration for enhanced CAR T cell function include manufacturing CAR T cells to secrete IL-7 as well as using synthetic IL-7 injections.^41^^,^^43^ Therefore, it may be potentially beneficial to use both dex to upregulate IL-7Rα on CAR T cells and IL-7 supplementation to improve CAR T cell persistence and function in patients undergoing CAR T cell therapy.

While dex is a potent immunosuppressive agent,^2^^,^^3^ prophylactic corticosteroid use in patients receiving CD19-CAR T cells for toxicity management results in low rates of CRS, delayed CRS onset, and similar rates of neuro-toxicity, without adversely affecting CAR T cell pharmacokinetics or efficacy outcomes.^5^^,^^44^ Moreover, the dose, timing and duration of GCs does not influence the clinical efficacy of CAR T cells in relapsed/refractory MM,^45^ and the cohort of patients treated with dex had better CAR T cell expansion and persistence as measured by prolonged B cell aplasia.^4^ Our data give potential context to these clinical observations and agree with previous preclinical studies showing that dex inhibits expansion and function of naive T cells, but not that of activated T cells.^16^ This research provides an opportunity to explore a new strategy for enhancing CAR T cell performance both during production and following infusion into patients.

## Materials and methods

### Cell lines

Human lymphoblastoid cells (LCLs) were generated as previously described by transforming peripheral blood mononuclear cells (PBMCs) with Epstein-Barr virus (EBV).^46^ LCL cells and Raji (ATCC, CCL-86) cells were maintained in RPMI 1640 with 10% heat-inactivated fetal bovine serum (FBS) (Hyclone, SH30070.03HI). KG-1a (ATCC, CCL-246.1) and SUP-B15 (ATCC, CRL-1929) cells were cultured in IMDM (Life Technologies, 12440-053) with 10% heat-inactivated FBS. MM.1S cells were purchased from ATCC and cultured in RPMI 1640 with 10% FCS. Epithelial ovarian cancer line derived from metastatic ascites OV-90 (ATCC, CRL-11732) was cultured in a 1:1 mixture of MCDB 105 medium (Millipore Sigma, M6395-1L) and Medium 199 (Gibco, 12350-039), adjusted to pH of 7.0 with sodium hydroxide, and final 20% FBS and 1× penicillin/streptomycin (Gibco, 15140122). To generate ffluc^+^GFP^+^ cell lines, KG-1a, SUP-B15, OV-90, and MM.1S cells were transduced with an eGFP-ffluc epHIV viral vector and sorted for 100% purity. Chinese Hamster Ovary cells (CHO) (CCL-61) from ATCC were transduced with hIL7_pIRESpuro3 plasmid to produce human recombinant IL-7 (CHO-IL7). CHO-IL7 cells were maintained in 50/50 DMEM/Ham’s F-12 (Corning, 10092CVR) with 10% heat-inactivated FBS and 10 μg/mL puromycin (InvivoGen, anti-pr-1).

### T cell isolation

Human HD blood was obtained from the City of Hope (COH) Blood Donor Center under protocols approved by the COH Institutional Review Board (IRB). To isolate PBMCs, blood was resuspended in PBS/2%FBS/EDTA and separated using Ficoll-Paque Plus (Cytiva, 17144002) density gradient centrifugation in SepMate50 tubes (StemCell Technologies, 85450), followed by two washes in PBS/2%FBS/EDTA. To isolate naive and memory T cells (T_n/mem_), PBMCs were resuspended in autoMACS Running Buffer (Miltenyi Biotech, 130-091-221) and up to 5 × 10^9^ cells were incubated with anti-CD14 microbeads (Miltenyi Biotech, 130-050-201) to eliminate monocytes and anti-CD25 microbeads (Miltenyi Biotech, 200-070-211) to eliminate regulatory T cells, for 30 min on ice. CD14^+^CD25^+^ cells were immediately depleted using the DEPLETES program on autoMACS Pro Separator (Miltenyi Biotech, 130-090-273) according to the manufacturer’s protocol. The unlabeled negative cells were resuspended in autoMACS Running Buffer and anti-CD62L microbeads (Miltenyi Biotech, 170-076-700), incubated for 20 min on ice, and immediately subjected to the POSSELD enrichment program on autoMACS according to the manufacturer’s protocol. For T_cm_ isolation, PBMCs were incubated with anti-CD14, anti-CD25, and anti-CD45RA (Miltenyi Biotech, 130-045-901) microbeads and depleted using the DEPLETES program on autoMACS. The unlabeled negative fraction was labeled with anti-CD62L microbeads and enriched with POSSELD program on autoMACS.

To generate EBV-specific T cells, purified HD T_cm_ (CD45RO^+^CD62L^+^) and T_em_ (CD45RO^+^CD62L^−^), cells were stimulated with 8,000 rad-irradiated autologous LCL cells at 4:1 (responder/stimulator) ratio weekly for 3 weeks. Resultant EBV-specific T cells were further expanded with a rapid expand method as previously described.^24^

### Generation of CAR T cells

Isolated HD PBMC and T_n/mem_ cells were stimulated with GMP Human T-expander CD3/CD28 Dynabeads (Dynal Biotech Cat#11141D) at a ratio of 1:3 (T cell:bead) overnight. Activated T cells were transduced with CD19R(EQ):CD28:ζ/EGFRt, CS1R(HL-CH3):41BB:ζ/EGFRt, or TAG72(HL-CH3):CD28tm-41BB-ζ(CO)/CD19t constructs at a multiplicity of infection of 1^11^^,^^18^^,^^23^ in RPMI 1640 containing 10% FBS, 5 μg/mL protamine sulfate (Fresenius Kabi, 22905), 50 U/mL rhIL-2 (Novartis Pharmaceuticals, NDC0078-0495-61), and 0.5 ng/mL rhIL-15 (CellGenix, 1013-050). After 7 days, CD3/CD28 Dynabeads beads were removed using a DynaMag-5 Magnet (Invitrogen, 12303D) and 7 × 10^5^ cells/mL were plated in RPMI 1640/10% FBS, 50 U/mL rhIL-2, and 0.5 ng/mL rhIL-15 to rest for 48 h. CAR T cells were cultured for 16–24 days before being used in *in vitro* experiments or being frozen in CryoStor (Biolife Solutions, 205102) for mouse experiments. For dex titration studies, 0.1 μM, 1 μM, or 10 μM dex (Sigma, D4902), reconstituted in ethanol, was added to the cell culture media in T75 flasks at day 9 only (for single dex dose) or days 9, 12, and 14 (for triple dex doses) and fresh media was added to maintain the cells at 0.7 × 10^6^/mL. Cells were analyzed on approximately day 16 or approximately day 23. IL-2 and IL-15 were supplemented every other day. TAG72-CAR T cells were generated and purified as previously described.^23^

### Antibodies and flow cytometry

For surface staining, cells were incubated with fluorochrome-conjugated monoclonal antibodies to CD3 (BD Bioscience, 563109, 557832), CD4 (BD Bioscience, 557852, 340133), CD8 (BD Biosciences, 348793), CD62L (BD Biosciences, 341012), CD127 (Biolegend, 351319), EGFR (Biolegend, 352906), LAG3 (LSBio, LS-B2237), PD1 (Invitrogen, 47-2799-42), TIM3 (R&D Systems, FAB2365P), CD45RA (BD Biosciences, 555488), CD45RO (BD Biosciences, 561137), CD45 (BD Biosciences, 340665), CD107a (BD Biosciences, 555800), CD45 (BD Biosciences, 340665), or CD19 (Life Technologies, MHCD1905). Cells were resuspended in fluorescence-activated cell sorting (FACS) buffer (HBSS [Gibco, 14175095], 2% FBS and NaN_3_ [Sigma, S8032]), and incubated with antibodies at 4°C in the dark. After washing cells with FACS buffer, DAPI (Invitrogen, D21490) was added for viability staining before analysis.

For intracellular staining, cells were stained with FVD Viability Viogreen (Thermo Fisher Scientific, 65-0866-18) at 1:1,000 dilution for 15 min at 4°C. Cells were washed with FACS buffer, fixed and permeabilized with Cytofix/Cytoperm Plus (BD Bioscience, 555028) for 20 min at 4°C, then stained with intracellular antibody for IFN-γ (BD Biosciences, 557643) at 4°C for 20 min. Flow cytometry was performed using MASCQuant Analyzer 10 (Miltenyi Biotech, 130-096-343) according to the manufacturer’s protocol. Flow cytometry results were analyzed using FCS Express 7 Research Edition.

### Degranulation

CAR T cells were cocultured with LCL cells at a 1:1 ratio, BD GolgiStop protein transport inhibitor (BD Bioscience, 554724), and antibody for CD107a (BD Bioscience, 555800) in the dark for 6 h at 37°C. KG-1a cells were used as negative control. CD107a expression was determined by flow cytometry.

### Cytokine production assay

CAR T cells were cocultured with LCL at a 1:1 ratio for 4 h at 37°C before adding Brefedlin A Golgi Plug (BD Bioscience, 555029). Cells were incubated at 37°C for 24 h before intracellularly stained for IFN-γ. KG-1a was used as a negative control.

### NanoString gene expression analysis

RNA preparation was performed according to the protocol for the nCounter FLEX system (NanoString Service). Raw data was processed with nCounter Advanced Analysis software (version 2.0.134) for pathway score analysis following manufacturer’s instructions. Pathways with similar changes (upregulation or downregulation) in PBMC-derived CD19-CAR T cells and Tn/mem-derived CD19-CAR T cells at indicated timepoints were included. For gene expression, raw data was first processed using nSolver 4.0 Analysis software (NanoString). Gene expression counts were normalized to positive controls and selected housekeeping genes (counts >100 and percent coefficient of variation [%CV] of >40 per the manufacturer’s suggestions). After normalization, gene expression between cells treated with dex (Dex^+^) versus without dex (Dex^−^) at specific timepoints were compared as follows: (Count_Dex+_ – Count_Dex–_)/Count_Dex–_ genes were grouped into an upregulated group (>0), a no change group (=0), and a downregulated group (<0). Genes in different groups within PBMC-derived CAR T cells and T_n/mem_-derived CAR T cells were excluded. To exclude genes with minor changes, the %CV cutoff value of 20 was applied based on the highest %CV from the selected housekeeping genes after normalization. The selections were done using R 3.6.1 and Excel (Microsoft).

### Mouse xenograft studies

Animal experiments were performed under protocols approved by COH Institutional Animal Care and Use Committee. For all studies, 5 × 10^5^ fflucGFP SUP-B15 cells, 2 × 10^6^ fflucGFP MM.1S cells, or 5 × 10^6^ fflucGFP OV-90 cells were i.v., i.t., or i.p. injected, respectively, into each 6- to 8-week-old NOD-scid IL2Rγnull (NSG) mice. To interrogate the effects of *ex vivo* dex on CD19-CAR or CS1-CAR T cells, 1 × 10^6^ PBMC-derived CAR T cells, generated with or without *ex vivo* dex, were injected i.v. into mice. For *in vivo* dex treatment comparisons, 1 × 10^6^ T_cm_-derived CD19-CAR T cells, PBMC derived CS1-CAR T cells, or TAG72-CAR T cells were injected i.v. (CD19 and CS1) or i.p. (TAG72). Dex was injected, at a 10 mg/kg dose, every 48 h based on dex’s biological half-life being 36–54 h, for the first month.^16^^,^^47^ Subsequent months had a single injection per week. Where indicated, IL-7-producing CHO cells were irradiated at 8,000 rads and injected i.p. into the mice. Tumor burden was monitored by live mice imaging using the LagoX optical imaging system (Spectral Instruments Imaging). For imaging, mice were injected i.p. with XenoLight D-luciferin potassium salt (PerkinElmer, 122799). Images were analyzed using Aura Imaging Software (Spectral Instruments Imaging). Tumor quantification was reported when all mice of a given group were still viable. Survival was dictated based on humane endpoint in all groups. Upon euthanasia, bone marrow, blood, and spleen were harvested for flow cytometry analysis. Retro-orbital bleeding techniques were used to collect blood from the mice and samples were run on flow cytometer for analysis.

### Statistics

Analysis was performed using Prism (GraphPad Software Inc.). The non-parametric Mann-Whitney test was applied to group comparisons and log rank (Mantel-Cox) was applied to Kaplan-Meier survival curves. p values of less than 0.05 were considered statistically significant.

### Study approval

NSG mice were purchased from The Jackson Laboratory and maintained by the Animal Resource Center at the COH. Mice were housed in a pathogen-free animal facility according to institutional guidelines. All animal studies were approved by the Institutional Animal Care and Use Committee (IACUC: 21034). HD blood was obtained from COH Blood Donor Center under protocols approved by COH IRB (IRB 09025).

## Data and code availability

The data generated in this study are available upon request from the corresponding author.

## Supplemental information

Document S1. Figures S1–S6

Document S2. Article plus supplemental information
