# Supplementary material for: Dexamethasone potentiates chimeric antigen receptor T cell persistence and function by enhancing IL-7Rα expression
Source: Mol Ther. 2023 Dec 22;32(2):527–39. doi: 10.1016/j.ymthe.2023.12.017 (PMC10861975; doi:10.1016/j.ymthe.2023.12.017)
Supplement: Document S1. Figures S1–S6 [file mmc1.pdf]

YMTHE, Volume *32*

Supplemental Information

Dexamethasone potentiates chimeric antigen receptor T cell persistence and function by enhancing IL-7Rα expression

Ashlie M. Munoz, Ryan Urak, Ellie Taus, Hui-Ju Hsieh, Dennis Awuah, Vibhuti Vyas, Laura Lim, Katherine Jin, Shu-Hong Lin, Saul J. Priceman, Mary C. Clark, Lior Goldberg, Stephen J. Forman, and Xiuli Wang

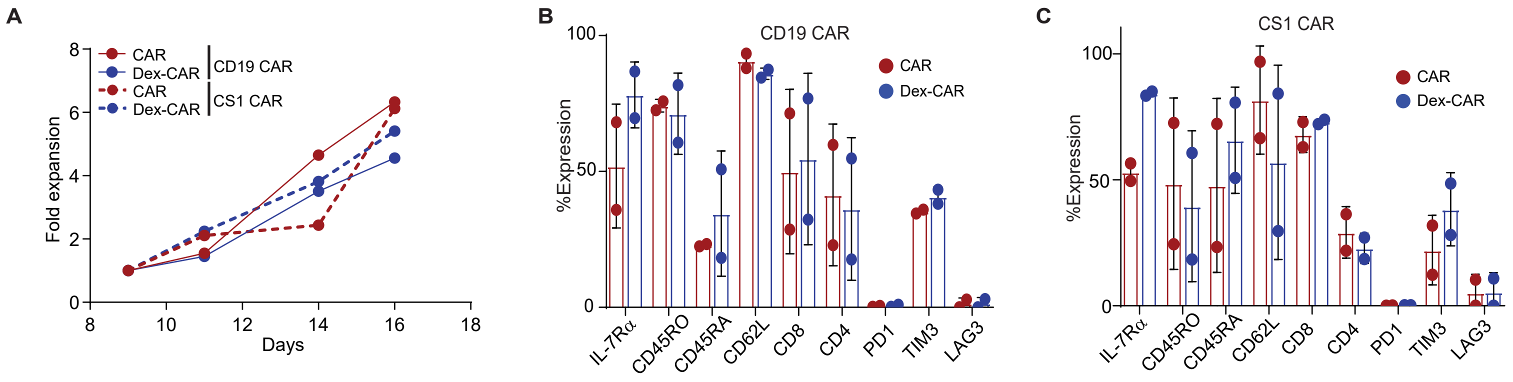

Figure S1. Dex does not affect CAR growth or phenotype regardless of CAR contructs. (A) Fold expansion of CD19- and CS1-CAR T cells from the same donor, in the presence of a single 1μM dex treatment. Phenotype of CD19- **(B)** and CS1- **(C)** CAR T cells from the same donor, in the presence or absence of a single 1μM dex treatment.

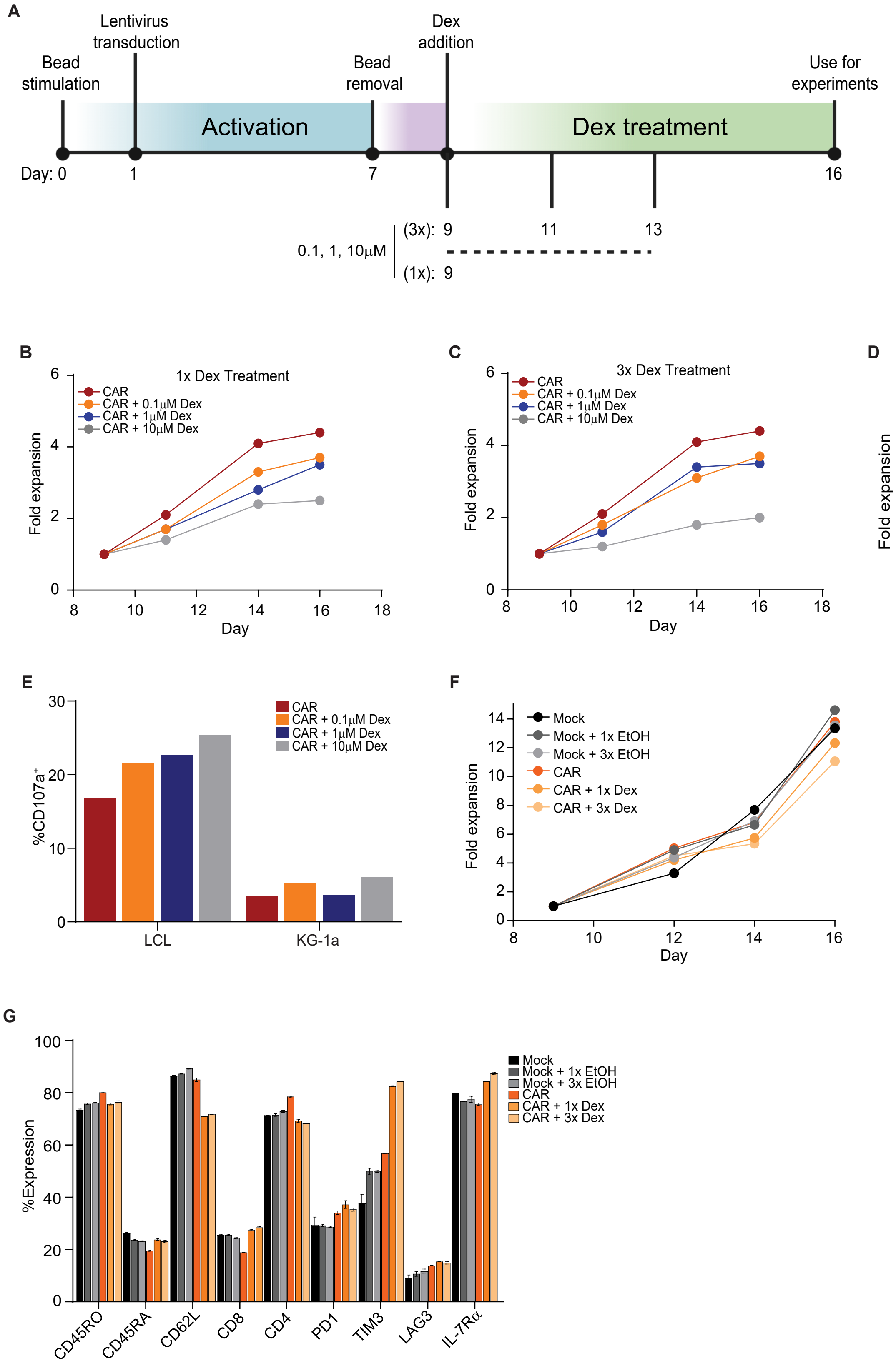

Figure S2. Multiple dex dosages of varying concentration do not affect CAR T cell growth, phenotype, or function. **(A)** PBMCs were activated with anti-CD3/anti-CD28 beads on day 0, transduced with lentivirus on day 1, followed by bead removal on day 7. CAR T cells were either treated with a single dose of varying concentrations of dex on day 9 or three doses on days 9, 11, and 13. Fold expansion of PBMC CD19-CAR T cells grown in the presence or absence of a single **(B)** or triple **(C)** 0.1μM, 1μM, or 10μM dex treatments. **(D)** Degranulation of PBMC CD19-CAR T cells, given different dex concentrations *ex vivo*, when co-incubated with CD19$^+$ LCL cells on day 16. KG-1a cells were a negative control. PBMC CD19-CAR T cell growth **(E-F)** or phenotype **(G)** on day 16, when given a single or triple 1uM dex dosages, or equal volume dex solvent ethanol.

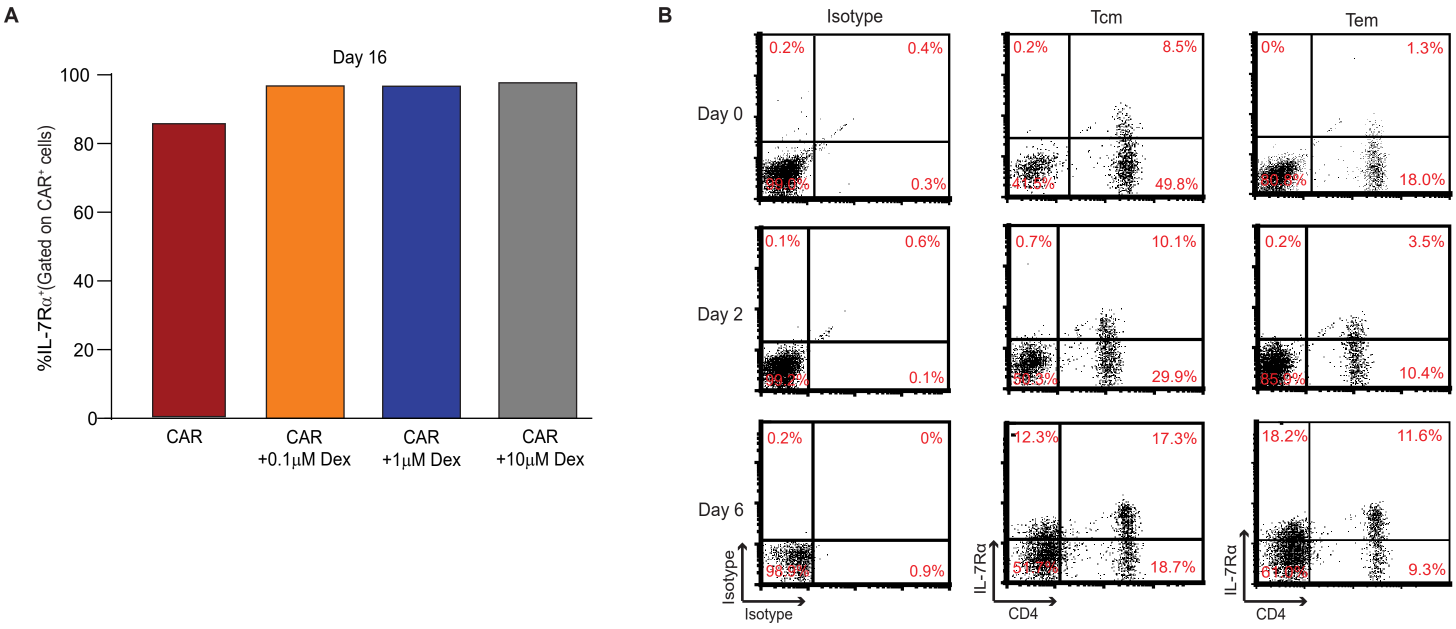

Figure S3. Expression of IL-7Rα is increased on dex-treated T cells. (A) Abundance of CAR+IL-7Rα+ CAR T cells when treated with a single dex dose of varying concentrations on day 16. (**B)** Phenotype of REM-expanded EBV-specific Tcm and Tem cells treated with a single 1μM dex dose, IL-7Rα levels were analyzed at different time points after dex treatment.

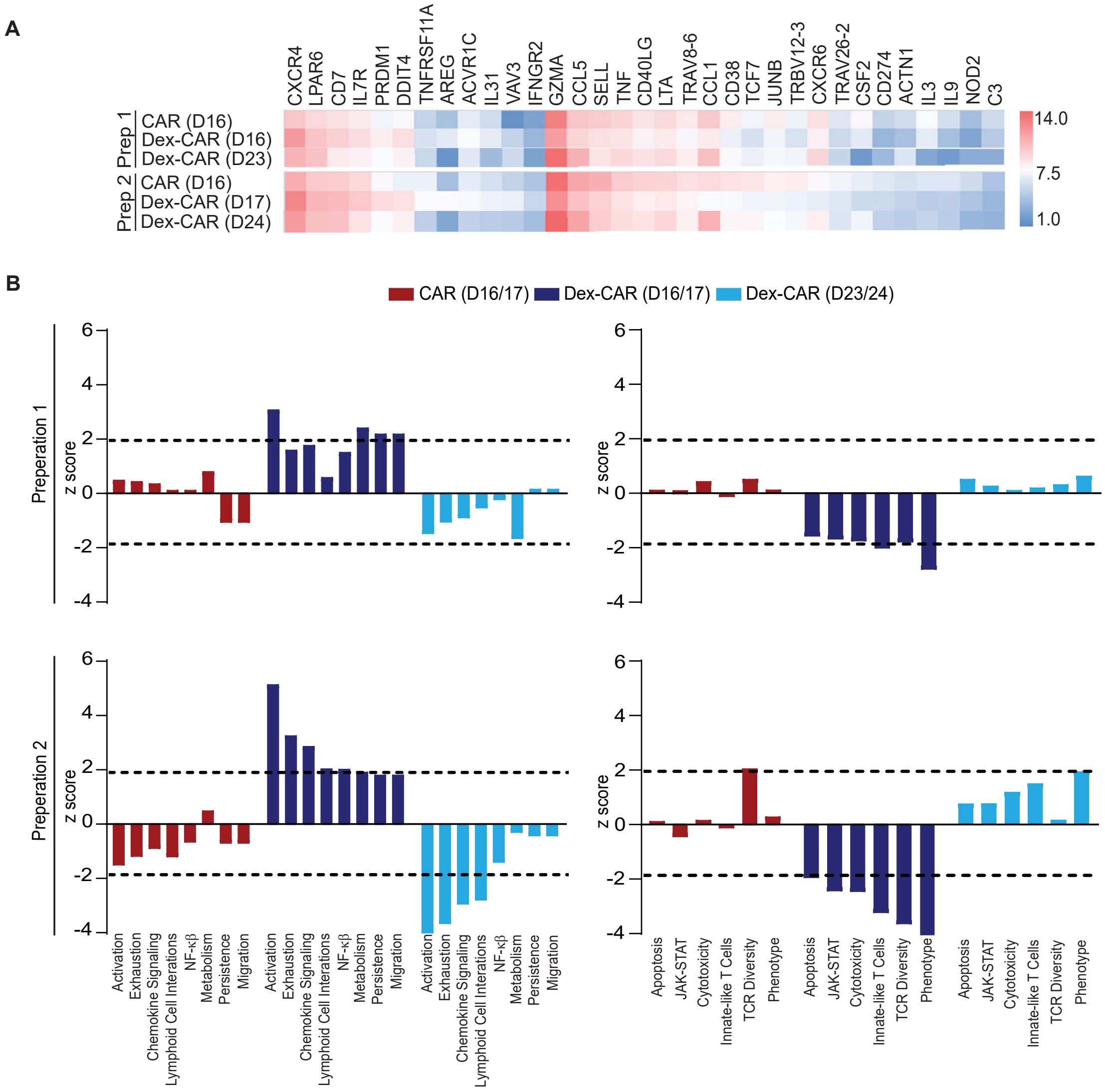

Figure S4. Dex regulates gene expression of PBMC- and Tn/mem-derived CD19-CAR T cells. CAR T cells treated with (dex-CAR) and without dex (CAR) were collected on day 16 and day 23 for PBMC-derived CAR T cells and day 17 and day 24 for Tn/mem-derived CAR T cells. Gene analysis was performed with NanoString technology. **(A)** Genes were first grouped into upregulated genes, downregulated genes, and genes with no response based on the changes of normalized gene expression counts in dex-CAR and CAR T cells. Only genes within the same groups in both PBMC-derived and Tn/mem-derived CAR T cells were included. Genes with minor changes were excluded based on the %CV cutoff value of housekeeping genes. The genes shown on the heatmap are on a log scale with base 2. **(B)** Pathway analysis was performed with NanoString. nCounter Advanced Analysis software provided by the manufacturer was applied for pathway score analysis. The dash lines indicated z = +/-1.96.

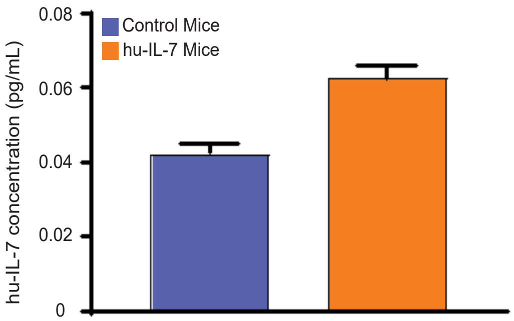

Figure S5. Serum concentration of human IL-7 in mice injected with huIL-7-secreting CHO cells. Serum from mice treated with IL-7-producing CHO cells was collected 24 hours post-IL-7-CHO cell injection. Serum from untreated mice was used as a control. Human IL-7 was measured with ELISA.

Acute lymphoblastic leukemia model

A

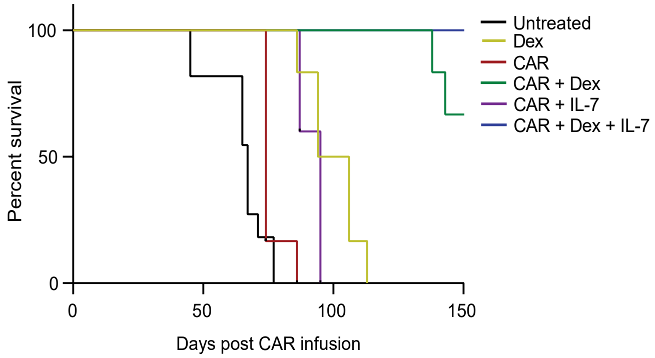

Ovarian cancer model

B

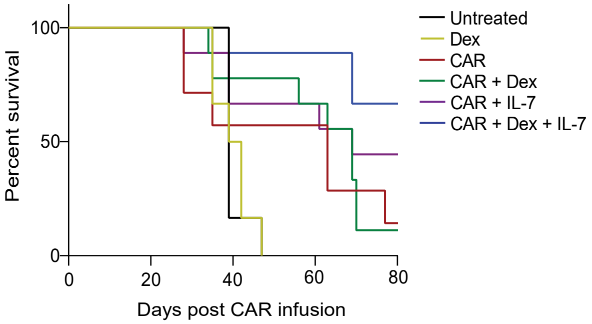

Figure S6. Dex and huIL-7, in combination with CAR T cells, prolongs *in vivo* survival in accumulative data. Accumulative data is shown for all experimental conditions in *in vivo* experiments, as previously shown, for both ALL (**A**) and ovarian (**B**) cancer models.
